# Blind blur assessment of MRI images using parallel multiscale difference of Gaussian filters

**DOI:** 10.1186/s12938-018-0514-4

**Published:** 2018-06-13

**Authors:** Michael E. Osadebey, Marius Pedersen, Douglas L. Arnold, Katrina E. Wendel-Mitoraj

**Affiliations:** 1 NeuroRx Research Inc, Montreal, 3575 Parc Avenue, Suite # 5322, Montreal, QC H2X 3P9 Canada; 2 Department of Computer Science, Norwegian University of Science and Technology, Teknologivegen 22, 2815 Gjovik, Norway; 3 Montreal Neurological Institute and Hospital, McGill University, 3801 University St, Montreal, QC H3A 2B4 Canada; 4 BrainCare Oy, Finn-Medi 1 PL 2000, 33521 Tampere, Finland

**Keywords:** Blur, Edges, Difference of Gaussian, Multi-scale representation, Local contrast feature image, Sharpness, Contrast

## Abstract

**Background:**

Rician noise, bias fields and blur are the common distortions that degrade MRI images during acquisition. Blur is unique in comparison to Rician noise and bias fields because it can be introduced into an image beyond the acquisition stage such as postacquisition processing and the manifestation of pathological conditions. Most current blur assessment algorithms are designed and validated on consumer electronics such as television, video and mobile appliances. The few algorithms dedicated to medical images either requires a reference image or incorporate manual approach. For these reasons it is difficult to compare quality measures from different images and images with different contents. Furthermore, they will not be suitable in environments where large volumes of images are processed. In this report we propose a new blind blur assessment method for different types of MRI images and for different applications including automated environments.

**Methods:**

Two copies of the test image are generated. Edge map is extracted by separately convolving each copy of the test image with two parallel difference of Gaussian filters. At the start of the multiscale representation, the initial output of the filters are equal. In subsequent scales of the multiscale representation, each filter is tuned to different operating parameters over the same fixed range of Gaussian scales. The filters are termed low and high energy filters based on their characteristics to successively attenuate and highlight edges over the range of multiscale representation. Quality score is predicted from the distance between the normalized mean of the edge maps at the final output of the filters.

**Results:**

The proposed method was evaluated on cardiac and brain MRI images. Performance evaluation shows that the quality index has very good correlation with human perception and will be suitable for application in routine clinical practice and clinical research.

## Background

Magnetic resonance imaging (MRI) system signal is sensitive to motion whereas patient motion is a common behaviour among subjects during brain and cardiac MRI acquisition sessions [1–3]. Major motion-related challenges include involuntary patient actions such as cardiac motion, respiratory motion and irregular heart beats. Other motion-related challenges include head motion and the movement of extremities. Steps taken to mitigate the effects of these motion-related challenges often requires trade-offs between MRI system operating parameters [4–6]. There is trade-off between high temporal resolution which account for cardiac and respiratory motion and large field of view which amplifies distortions. Concern for the comfort of elderly patients, unpredictable actions of very young children and the mentally unstable patients calls for compromise between signal-to-noise ratio, image resolution and length of scan time.

These challenges introduce distortions such as noise, bias fields and blur which limits the acquisition of high quality image. The focus of this report is on blur. Blur can be considered a unique type of distortion in comparison to noise and bias fields. Blurred boundaries is the consequence of partial volume effect in regions where the boundary between two different tissues is not orthogonal to image slice [7, 8]. Beyond the acquisition stage blur can be introduced into an image as a result of postacquisition processing and the manifestation of pathological conditions. Reported MRI findings in patients with focal cortical dysplasia (FCD) is the cortical thickening and blurring of the grey-white matter boundary [9, 10]. Post acquisition processing methods such as Karhunen–Loeve transform and the use of linear filters for de-noising of cardiac and brain MRI images are known for the blurring of edges with the consequent loss of diagnostic information [11, 12].

Blur, like all distortion processes, is uniformly propagated throughout an image. However, the effect of blur is not uniformly distributed in MRI images because the human anatomy is structurally heterogeneous. Blur weakens the strength of edges which define the visibility of details within an image [13]. Blur erodes the texture features that characterize smoothly varying regions such as the cardiac ventricles and the brain white matter. It causes loss of sharpness in the high density of edges that describe the cortical grey matter region and reduces the contrast between the different anatomical structures [14].

Blur assessment is, and will continue to be an active research area in the image processing community because the reliability of metrics derived from MRI images for the diagnostic evaluation of cardiac and neurological diseases, to a large extent, is dependent on edge information. Edge information is strongly related to the level of blur in an image. Several physiological parameters are based on edge-based metrics derived from MRI images. The physiological parameters include cardiac ejection fraction, myocardial wall motion, blood flow velocity, myocardial perfusion, whole brain volume measurement, whole brain atrophy, white matter atrophy and cortical grey matter atrophy [15–19].

Most current blur assessment algorithms are designed for a general class of images with focus on consumer electronics such as digital cameras, television, video and mobile devices. Generally, the algorithms begins with the extraction of an edge map from the test image. Blur quality index is derived after the edge map is further analyzed in one or combinations of the spatial domain, frequency domain or multi-resolution decomposition. In this report we categorize current blur assessment methods into recent and earlier contributions. Recent contributions include the reports in [20–24]. Earlier contributions include the reports in [25–30]. It is not possible to list all the current contributions. However, we will describe the unique design features which distinguish the aforementioned recent contributions.

The concept of increased dynamic range was introduced in [20]. Increasing the dynamic range of generated contrast maps significantly improve blur prediction. Another report measure the blurriness in an image from the steepness of probability density function. The probability density function models the histogram of discrete cosine transform coefficients of edge maps [21]. Color, edge, and structural information is the technique used to discriminate images with different levels of blur in [22]. Exact Zernite moments which reflects human visual characteristics was extracted from test images in [23]. The exact Zernite moments are combined with contrast information from gradient magnitude to measure the level of blur in the image. Changes in structural information resulting from blurriness was encoded with orthogonal moments and visual saliency model in [24].

One of the few contributions focused on medical images is the edge sharpness assessment by parametric (ESAP) modeling [23]. Sharpness assessment in ESAP begins with manual selection of region of interest from edge map extracted from the test image. The intensity level of edge pixels that are appropriate to describe edge sharpness are read and fitted with a sigmoid function. Sharpness quality score is computed from the parameters of the sigmoid function. Another report is based on the Moran statistics [31]. Moran statistics, originally proposed to estimate noise level, is a function of the spatial autocorrelation of mapped data [32]. The peak ratio of Moran’s histogram quantifies the degree of image blurring based on the notion that the quantity of image blurring is dependent upon the ratio between the processed peak of Moran’s histogram and the original image.

The region-of-interest incorporated in ESAP is a novelty. However the authors acknowledge that ESAP may not correlate with human visual perception. Furthermore, manual selection of the region-of-interest limits its application where large volumes of MRI data are processed. The versatility of the report based on Moran statistics is limited because it is a full-reference method.

In this report we propose a new approach to assess blur distortion in MRI images. The concept behind blur quality evaluation is the existence and persistence of edge information at different image resolutions [21]. Across increasing Gaussian scales, edges in higher quality images have higher persistence than lower quality images. Blur quality is derived from the relationship between three image features. The proposed method incorporates human visual characteristics. The test image is simultaneously fed into two parallel difference of Gaussian (DOG) filters which operate with different parameters at multiscale representation. The different parameters constrains one filter to successively attenuate edges and the other filter to highlight edges over the same fixed range of multiscale representation. Image quality score variable is the distance between the features extracted from the output of each filter at the end of multiscale representation.

The next section describes the methods for our proposed quality assessment "Experiments" section describe the objective and the subjective performance evaluation experiments of the proposed quality metric. Results from the experiment are displayed and discussed in "Results" and "Discussion" sections, respectively. Challenges, limitations and future work is in "Challenges, limitations and future work" section. "Conclusion" section concludes this report.

## Methods

**Fig. 1:**
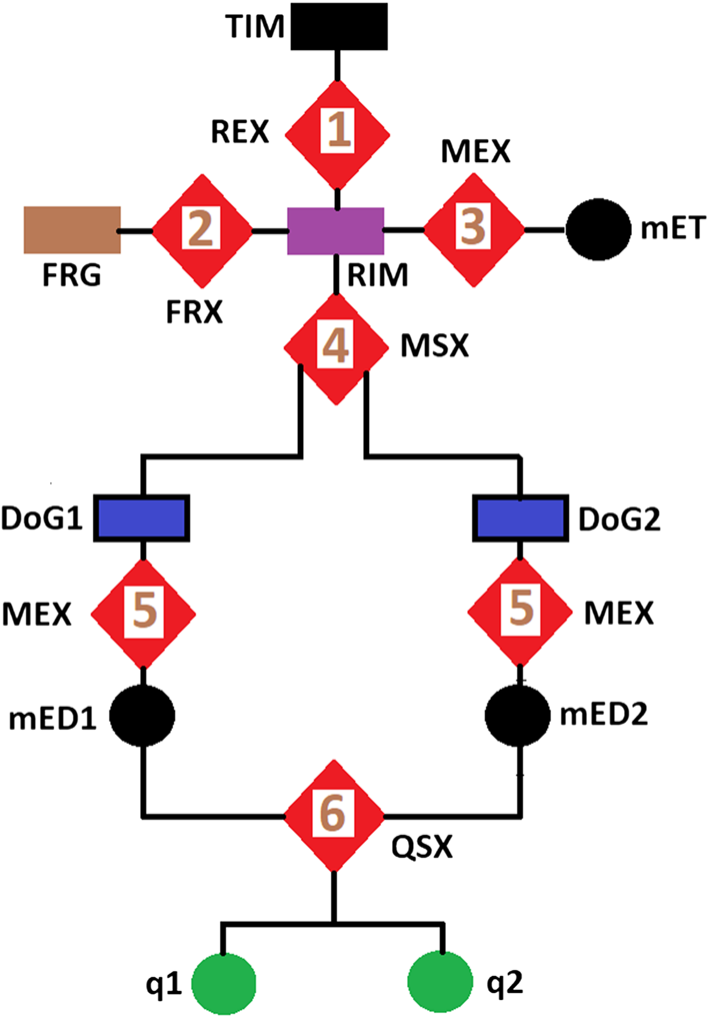
Flow chart of the proposed method for the assessment of blur in MRI images

**Fig. 2:**
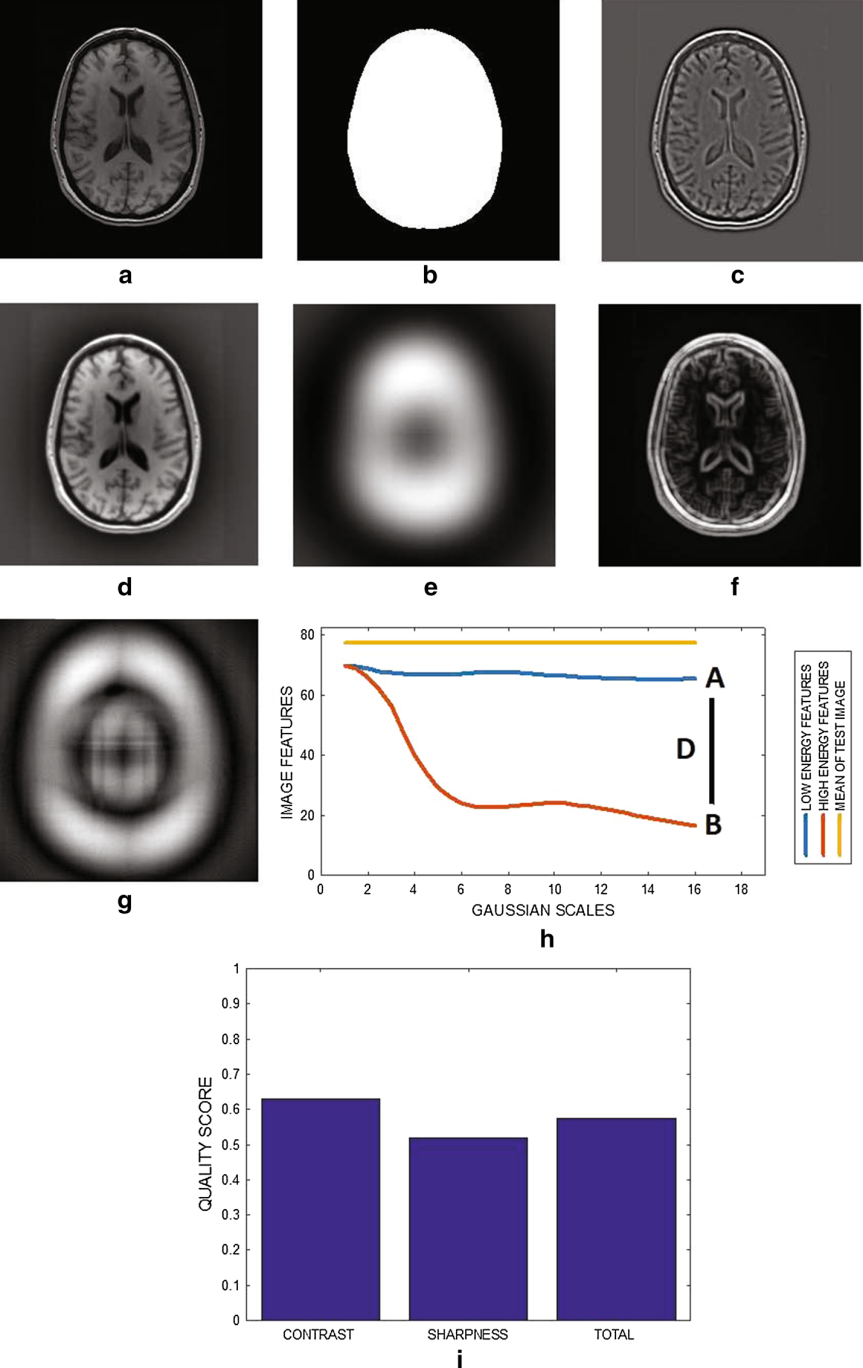
The implementation of blind blur assessment in MRI images. **a** The test image has its pixel intensity level rescaled to lie between 0 and 255. **b** Foreground of the test image in **a** is extracted. **c** The identical edge map from the initial parameters of the low and high energy difference of Gaussian filters. **d** The output image of the low energy filter at the conclusion of the multiscale representation. **e** The output image of the high energy filter at the conclusion of the multiscale representation. **f** The edge map extracted from the image in **d**. **g** The edge map extracted from the image in **e**. **h** Variation of image features from the output of the low and high energy filters at different Gaussian scales. **i** The predicted contrast, sharpness and total blur quality scores based on the analysis of the plot in **h**

The flow chart in Fig. 1 and the images in Fig. 2 explains the six sequential steps to implement the proposed blur assessment method. Three symbols used in the flowchart are diagonals, circles and rectangles. A diagonal represents each step in the implementation of the proposed method. The circles are the output of numeric computations and the rectangles are images. The black, brown, purple and blue rectangles are the original image **TIM**, foreground image **FRG**, rescaled original image **RIM** and difference of Gaussian filtered images **DoG1**, **DoG2**, respectively.

### Step 1: intensity standardization

The original image is rescaled **REX** to produce a new image $I_{d}$ shown in Fig. 2a with intensity levels that lies between 0 and 255. The algorithm standardizes the intensity of all test images by rescaling their intensity levels to lie within the same fixed range. Intensity standardization ensures the standardization of contrast measures. Standardization of contrast measures makes it possible to compare predicted blur assessment indices for different images and images with different contents.

### Step 2: extraction of foreground

The foreground region shown in Fig. 2b was extracted **FRX** from the rescaled original image shown in Fig. 2a to determine the region covered by the anatomical structures within the image grid. The area of foreground region is required to compute feature descriptors in subsequent steps of the algorithm. There are three successive stages within the foreground extraction step. The first step is global threshold set at the first moment of the rescaled orginal image. The output of the global threshold is a binary image. The global threshold is followed by morphological hole filling operation of the binary image. After the hole filling operation, there is morphological cleaning operation of the same binary image. In the cleaning operation, small regions $\ge 800$ pixels that are unfilled in the hole filling operation are detected and eliminated.

### Step 3: compute image feature

The mean **mET** of the rescaled original image is computed **MEX** from the indices of foreground pixels extracted in step 2.

### Step 4: parallel multiscale DoG filtering

Two duplicate copies of the rescaled original image are generated. Each duplicate is separately and simultaneously convolved with two difference of Gaussian filters (DOG). The filters, $DoG_{(\sigma _{1},\sigma _{2})}(x,y)$ and $DoG_{(\sigma _{3},\sigma _{4})}(x,y)$ are defined as:

1 $$\begin{aligned} DoG_{(\sigma _{1},\sigma _{2})}(x,y)= & {} \left( G_{\sigma _{1}}(x,y) - G_{\sigma _{2}}(x,y)\right) \nonumber \\ DoG_{(\sigma _{3},\sigma _{4})}(x,y)= & {} \left( G_{\sigma _{3}}(x,y) - G_{\sigma _{4}}(x,y)\right) \end{aligned}$$

where $\sigma _{1},$ $\sigma _{2},$ $\sigma _{3}$ and $\sigma _{4}$ are the widths of the Gaussian filter $G_{\sigma }(x,y):$

2 $$\begin{aligned} G(x,y)=\frac{1}{\sqrt{2\pi }\sigma }\exp \left( -\frac{x^{2}+ y^{2}}{2\sigma ^{2}}\right) \end{aligned}$$

The motivation behind the use of DOG filter is its efficient application in edge detection for feature enhancement, blob detection, face detection and quality evaluation [33–36]. The DOG filter was implemented using the matlab code available in [37, 38]. Human visual system characteristics are incorporated into the algorithm by tuning each DOG filter to different parameters and for multiscale representation **MSX** of the rescaled original image. The parameters $\theta _{1},$ $\theta _{2}$ for each filter are defined as:

3 $$\begin{aligned} \theta _{1}&= \{\sigma _{1}, (\sigma _{1} + \sigma _{2})\}, \quad \sigma _{1} = 1, \quad \sigma _{2}=\{1,2,3,\ldots ,L\} \nonumber \\ \theta _{2} & = {} \{ \sigma _{3}, (\sigma _{3} + \sigma _{4})\} \quad \sigma _{3} = \{1,2,3,\ldots ,L\}, \quad \sigma _{4}=1 \end{aligned}$$

where *L*, the range of the multiscale representation, is defined as:

4 $$\begin{aligned} L=\frac{\sqrt{d1+d2}}{2} \end{aligned}$$

where *d*1 and *d*2 are the row and the column dimensions of the image, respectively. The output of each filter, at each scale of the multiscale representation are denoted **DoG1** and **DoG2** in the flow chart shown in Fig. 1.

Based on the parameter definitions in Eq. 3, the initial output from both filters are identical because, the initial parameters of both filters are equal:

5 $$\begin{aligned} \theta _{1}=\theta _{2}=\{1, 2\}. \end{aligned}$$

The initial output from one of the filters is shown in Fig. 2c. In subsequent multiscale representations each filter is tuned to different parameters. The first filter successively increases the level of details while the second filter successively attenuates the level of details. Based on these characteristics the filters are referred to as low and high energy DOG filters, respectively. The output from each filter, at the end of the multiscale representation, are displayed in Fig. 2d, e.

### Step 5: extract edge map

At each scale of the multiscale representation, an edge map is extracted from each filter. The mean (**mED1**), (**mED2**) of the edge map from each filter is also computed (**MEX**) from the indices of the foreground pixels. The edge map is the local contrast feature image extracted from the output of the filter using local contrast filters. The edge map extracted from the output (shown in Fig. 2d, e) of each filter are displayed in Fig. 2f, g, respectively. The extracted edge information is sensitive to the size of filter. Heuristic approach was adopted to determine the appropriate filter size. During the performance evaluation of the algorithm, it was observed that the use of $3 \times 3$ and $7 \times 7$ filter sizes does not predict quality score which correlated with subjective evaluation by human observers. Specifically, filter size of $3 \times 3$ underestimate image quality while filter size of $7 \times 7$ overestimate image quality. We recommend fixed filter size of $5 \times 5$ for images with dimensions $256 \times 256$ and $512 \times 512,$ respectively.

### Step 6: compute blur quality index

The level of blur is evaluated from the relationship between image features in each DOG filter. The plot in Fig. 2h show how the first moment of the edge map extracted from each DOG filter vary with different multiscale representations. The blue and red colored plots are for the first and second filters, respectively. Points A and B on the plot represent the first moment of each edge map at the conclusion of the multiscale representation. The distance between A and B is D. The yellow colored plot represents the fist moment of the rescaled original image. The fist moment of the rescaled original image serves as a reference for measuring the persistence of edges in each filter at different Gaussian scales. There are three image features of interest. The first feature of interest is the mean of the rescaled original image $\mu _{I_{d}}.$ The second and third features of interest are the first moments $\mu _{C_{A}},$ $\mu _{C_{B}}$ of the edge map extracted from each filter at the conclusion of the multiscale representation.

The followings hold:

6 $$\begin{aligned}\mu _{C_{B}} & \le \mu _{C_{A}},\nonumber \\ \mu _{C_{A}} & \le \mu _{I_{d}}, \nonumber \\ \mu _{I_{d}} & \le (\mu _{I_{d}} + \mu _{C_{B}}). \end{aligned}$$

Hereafter, we analyze the plot of the multiscale representation and show that the plot can be used to predict **QSX** quality index (Fig. 2i) for ideal, extremely degraded and real MRI images.

#### 1 Ideal image

An ideal MRI image is piecewise constant [39]. The edge map in an ideal MRI image is optimized. There are no more details to highlight by the first DOG filter. At the end (point **A** in Fig. 2h) of the multiscale representation, the final output image $I_{A}$ from the first DOG filter closely approximates the rescaled original image $I_{d}.$ Therefore,

7 $$\begin{aligned} I_{A}& \approx {} I_{d}, \nonumber \\ C_{A} & \approx {} I_{d}, \nonumber \\ \mu _{C_{A}} &\approx {} \mu _{d}. \end{aligned}$$

The second DOG filter successively attenuates edges in the ideal image. At the end (point **B** in Fig. 2h) of the multiscale representation, there is almost complete depletion of details in the rescaled original image. Therefore,

8 $$\begin{aligned} \mu _{C_{B}}\approx 0. \end{aligned}$$

The distance $D_{L_{1}}$ between the mean of the edge map at **A** and the mean of the edge map at **B**:

9 $$\begin{aligned} D_{L_{1}} \approx \Vert \mu _{C_{A}} - \mu _{C_{B}} \Vert = \mu _{C_{A}}. \end{aligned}$$

#### 2 Extremely degraded image

There is absence of details or very sparse details in an extremely degraded MRI image. There are no more details to highlight. At the end of the multiscale representation, the first DOG filter replicates the extremely degraded image. Therefore,

10 $$\begin{aligned} \mu _{C_{A}}\approx 0. \end{aligned}$$

The second DOG filter will completely erode the sparse details in the extremely degraded image. Therefore,

11 $$\begin{aligned} \mu _{C_{B}}\approx 0. \end{aligned}$$

The distance $D_{L2}$ between the mean of the edge maps at the output of each filter is:

12 $$\begin{aligned} D_{L2} = \Vert \mu _{C_{A}} - \mu _{C_{B}} \Vert \approx 0. \end{aligned}$$

#### 3 Real image

We postulate that the distance between the edge maps at the output of the low and the high energy filters is a useful variable for predicting the blur index of an MRI image. The quality index for a real MRI image will lie between the quality index of an extremely degraded image and the quality index of an ideal image:

13 $$\begin{aligned} 0 \le (D_{L1},D_{L2}) \le 1. \end{aligned}$$

The contrast between the edge and the non-edge regions in the rescaled original image is the contrast quality score. The contrast quality score $q_{1}$ is determined by normalizing the distance $D_{L}$ with the mean $\mu _{I_{d}}$ of the image:

14 $$\begin{aligned} q_{1}=\frac{\Vert \mu _{C_{A}} - \mu _{C_{B}}\Vert }{\mu _{I_{d}}}, \quad (\mu _{C_{A}} - \mu _{C_{B}}) \le \mu _{I_{d}} \end{aligned}$$

where $(\mu _{C_{A}} - \mu _{C_{B}}) \le \mu _{I_{d}}$ expresses the condition for the validity of *q*1. The sharpness of the rescaled original image is the sharpness quality score.

The sharpness quality score $q_{2}$ is determined by normalizing the distance $D_{L}$ with the $(\mu _{I_{d}} + \mu _{C_{B}}){:}$

15 $$\begin{aligned} q_{2}=\frac{\Vert \mu _{C_{A}} - \mu _{C_{B}}\Vert }{\mu _{I_{d}} + \mu _{C_{B}}}, \quad (\mu _{C_{A}} - \mu _{C_{B}}) \le (\mu _{I_{d}} + \mu _{C_{B}}) \end{aligned}$$

where $(\mu _{C_{A}} - \mu _{C_{B}}) \le (\mu _{I_{d}} + \mu _{C_{B}})$ expresses the condition for the validity of *q*2.

The total quality score *Q* is the average of the contrast and sharpness quality scores:

16 $$\begin{aligned} Q=\frac{q_{1}+ q_{2}}{2}. \end{aligned}$$

The difference in the computed values between the contrast and sharpness quality scores is the second term $\mu _{C_{B}}$ in the normalizing constant in Eq. 15. The choice of $\mu _{I_{d}}$ and $(\mu _{I_{d}} + \mu _{C_{B}})$ as the normalizing constant in Eqs. 14 and 15 is based on the expression in Eq. 13. The normalizing constant ensures that the blur quality index is defined within a lower and upper limit $\{0 \le (q_{1},q_{2}) \le 1\}.$

## Experiments

Performance evaluation of our proposed method was carried out using brain and cardiac MRI volume data. The brain MRI volume data were provided by NeuroRx research Inc. (https://www.neurorx.com), BrainCare Oy. (http://braincare.fi/) and the Alzheimer’s disease neuroimaging initiative (ADNI) (http://www.adni.loni.usc.edu). The cardiac MRI volume data were short axis MRI provided by Department of Diagnostic Imaging of the Hospital for Sick Children in Toronto, Canada (http://www.sickkids.ca/DiagnosticImaging/index.html). The cardiac MRI data were originally used as test data in the report [40].

There are 1200 slices from 25 short axis cardiac MRI volume data. The dimension of each cardiac slice is $256 \times 256$ pixels along the long axis. The slices of the brain MRI volume data are 500 T2, 250 T1 and 300 Fluid Attenuated Inversion Recovery (FLAIR) images. The brain MRI slices from NeuroRx and ADNI have dimension $256 \times 256$ pixels. The data from BrainCare have dimension $448 \times 390$ pixels.

The new blur assessment method was implemented in the MatLab computing environment. Gaussian blur and motion blur at different levels were artificially induced on the test data. Gaussian blur was simulated by convolving a slice with rotationally symmetric low pass filter of width *w*, $\{w:3< w < 15\}$ pixels. The range of the filter size was scaled from level 1 to level 15. The motion blur was induced on a slice by convolving it with a special filter which approximates the linear motion of a camera. The linear motion is described by two parameters, the linear distance in pixels and the angular distance in degree. Both parameters were scaled from 1 to 15 in unit step.

The performance of our proposed method was evaluated objectively and validated subjectively in four categories of experiments, The categories of the experiments are Cardiac MRI, T2, T1 and FLAIR brain MRI. Subjective evaluation was facilitated using **QuickEval** [41], a web-based tool for psychometric image evaluation provided by the Norwegian Colour and Visual Computing Laboratory (http://www.colourlab.no/quickeval) at the Norwegian University of Science and Technology, Gjovik, Norway. The observers are one radiologist and one medical imaging professional. The observers assigns a score between 0 and 100, in steps of 1, to each slice. Each score assigned by the observer is divided by 100 to ensure that the subjective and objective scales are in the same range. Each observer was first presented with an undistorted version of an MRI slice, followed by increasing distortion levels of the original slice. The distorted levels are 5, 10 and 15. The mean opinion score (MOS) was used in the validation studies because it is popular and simple to implement [42]. The relationship between the score predicted by our proposed method and the subjective scores assigned by human observers was computed using the spearman rank correlation coefficient [43].

## Results

### Objective evaluation

#### Brain MRI without perceived distortion

**Fig. 3:**
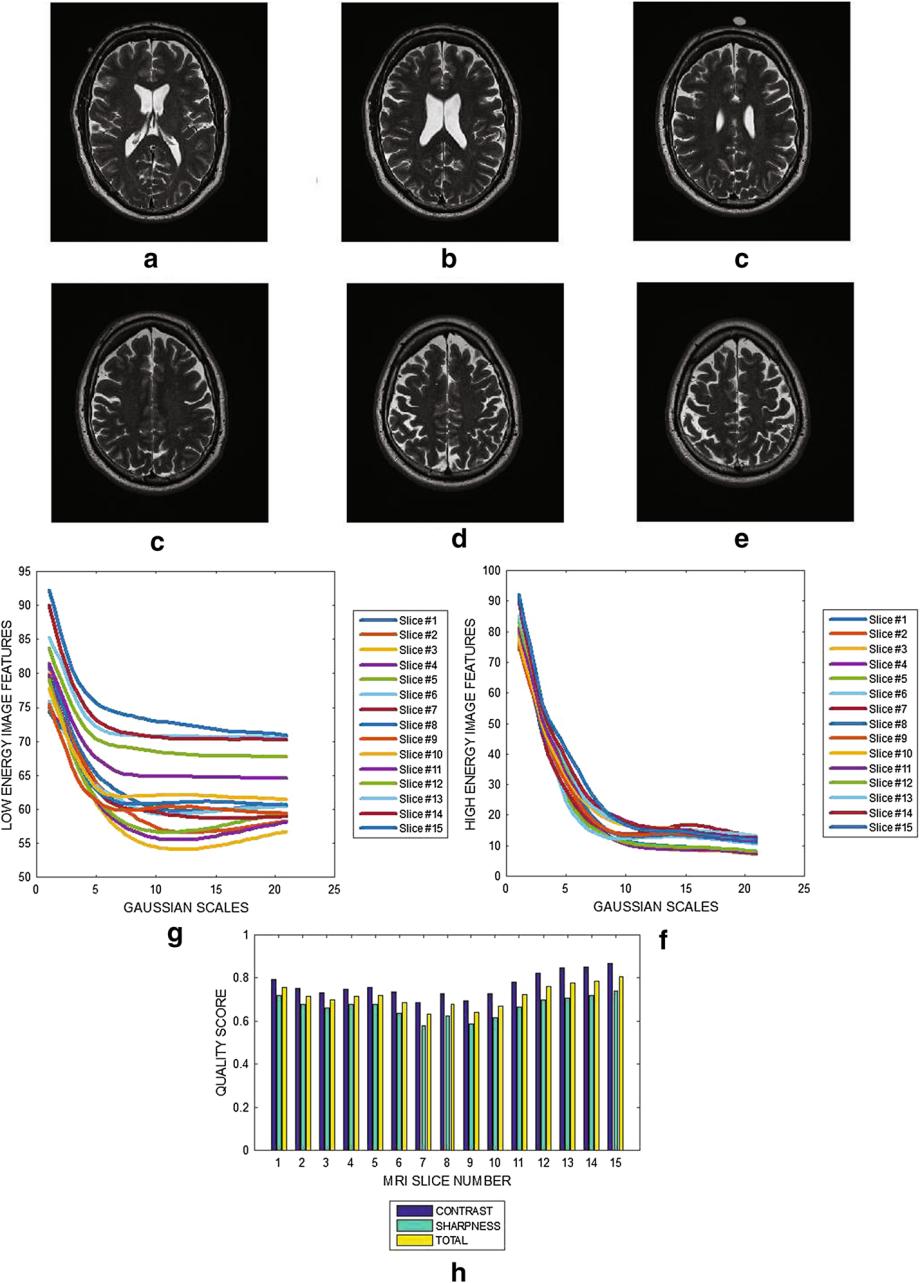
Six slices with slice numbers **a** 1, **b** 4, **c** 8, **d** 11 , **e** 13 and **f** 15 from T2 brain MRI volume data from BrainCare. **g** Variation of image features of each slice from the output of the low energy Gaussian filter at different Gaussian scales. **h** Variation of image features of each slice from the output of the high energy Gaussian filter at different Gaussian scales. **i** Contrast, sharpness and total quality scores of 15 slices from the MRI volume data

Six slices from a T2 weighted brain MRI volume data are shown in Fig. 3a–f. The variation of image features (mean of the edge maps) at different Gaussian scales for the low and the high energy DOG filters are shown in Fig. 3g, h. The plot in Fig. 3i show the contrast, sharpness and the total quality scores for 15 slices from the MRI volume data. The results shows that the slices in same MRI volume data have different levels of blur. The minimum and maximum blur levels are $\approx 0.7$ and $\approx 0.85,$ respectively.

#### Cardiac MRI without perceived distortion

**Fig. 4:**
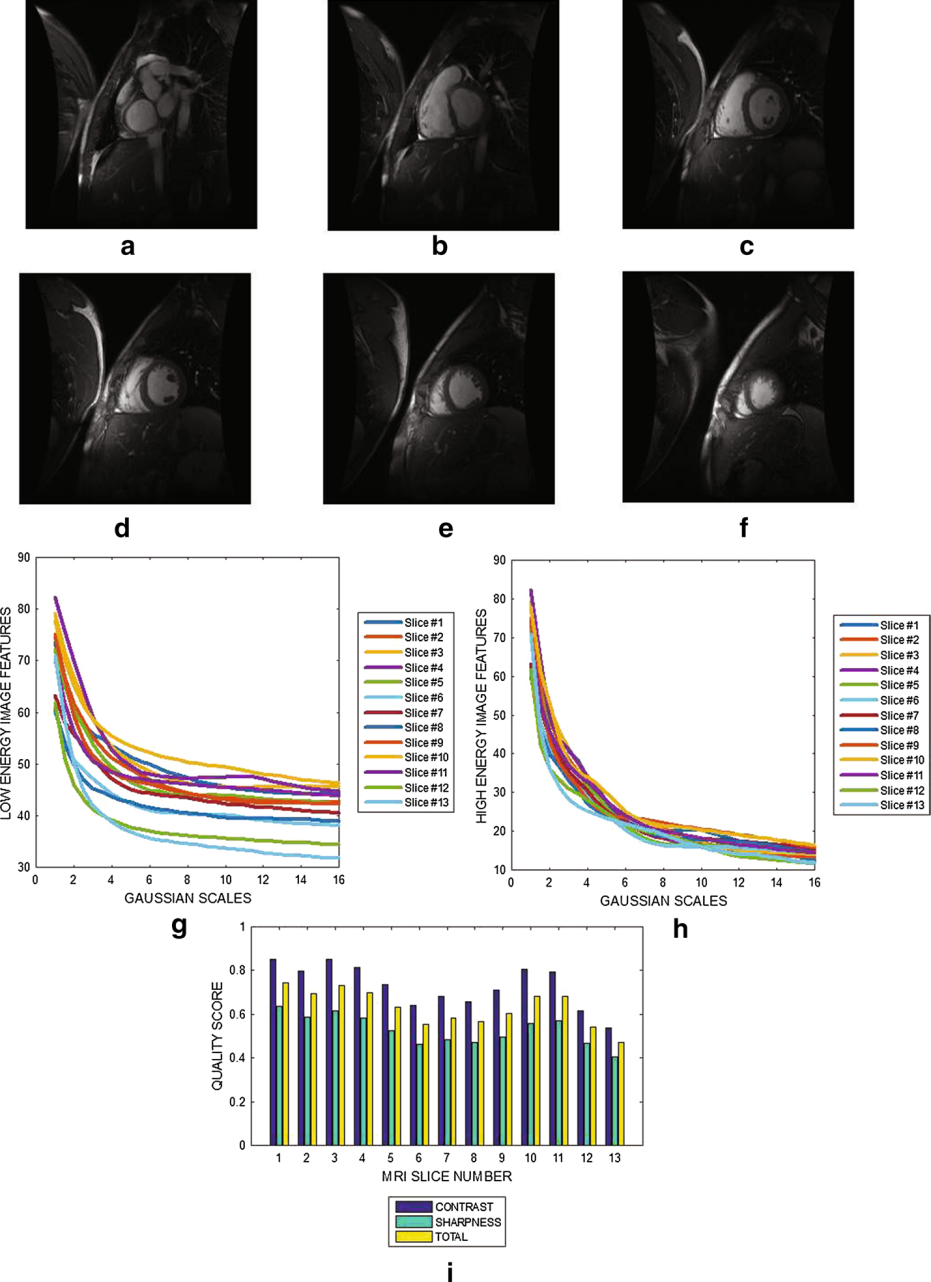
Six slices with slice numbers **a** 1, **b** 3, **c** 5, **d** 7 , **e** 9 and **f** 11 from short axi MRI volume data from Department of Diagnostic Imaging of the Hospital for Sick Children in Toronto. **g** Variation of image features of each slice from the output of the low energy Gaussian filter at different Gaussian scales. **h** Variation of image features of each slice from the output of the high energy Gaussian filter at different Gaussian scales. **i** Contrast, sharpness and total quality scores of 15 slices from the MRI volume data

Six slices from a cardiac MRI volume data are shown in Fig. 4a–f. Figure 4g, h show the variation of image features at different Gaussian scales for the low and the high energy DOG filters. The plot in Fig. 4i show the contrast, sharpness and the total quality scores for 13 slices from the MRI volume data. Blur levels in the cardiac slices contained in the same volume data vary from 0.45 to 0.83.

#### Gaussian blur

**Fig. 5:**
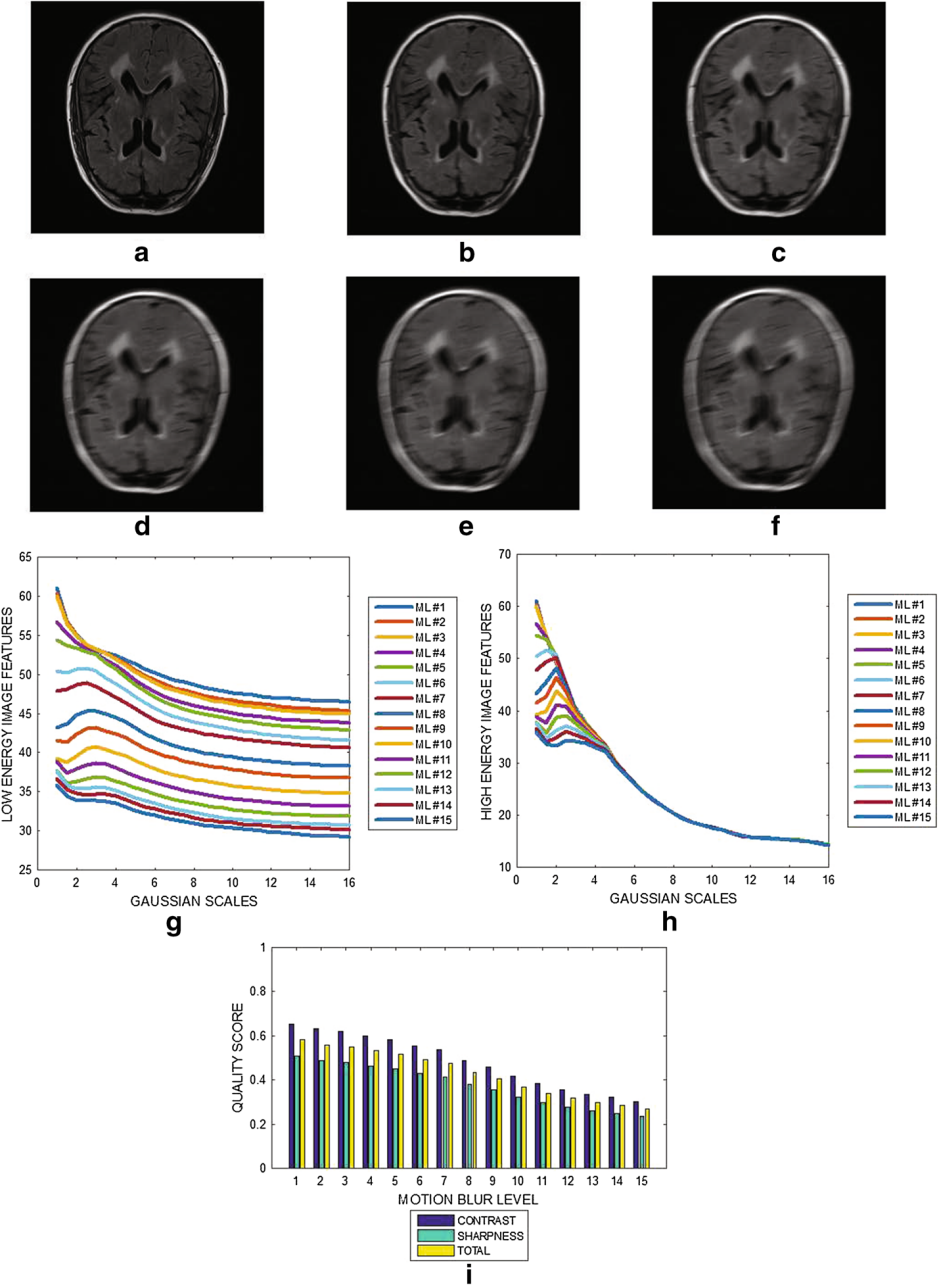
**a** FLAIR brain MRI slice from ADNI and its degraded versions at motion blur levels **b** 4, **c** 7, **d** 10, **e** 13 and **f** 15, **g** variation of image features of each slice from the output of the low energy Gaussian filter at different Gaussian scales. **h** Variation of image features of each slice from the output of the high energy Gaussian filter at different Gaussian scales. **i** Contrast, sharpness and total quality scores for different levels of motion blur

The image in Fig. 5a is a slice from a FLAIR brain MRI volume data. The images in Fig. 5b–f are the same image in Fig. 5a but were blurred with Gaussian filter at levels 4, 7, 10, 13 and 15, respectively. Given specific level of Gaussian blur, the variation of the image features at different Gaussian scales for the low and the high energy DOG filters are displayed in Fig. 5g, h, respectively. The contrast, sharpness and the total quality scores for Gaussian blur levels that vary from 0 to 15 are shown in Fig. 5i. In the absence of distortion, the blur level is 0.65. Increasing blurriness decreases the quality of the image from 0.65 to 0.35, for blur level of 15.

**Fig. 6:**
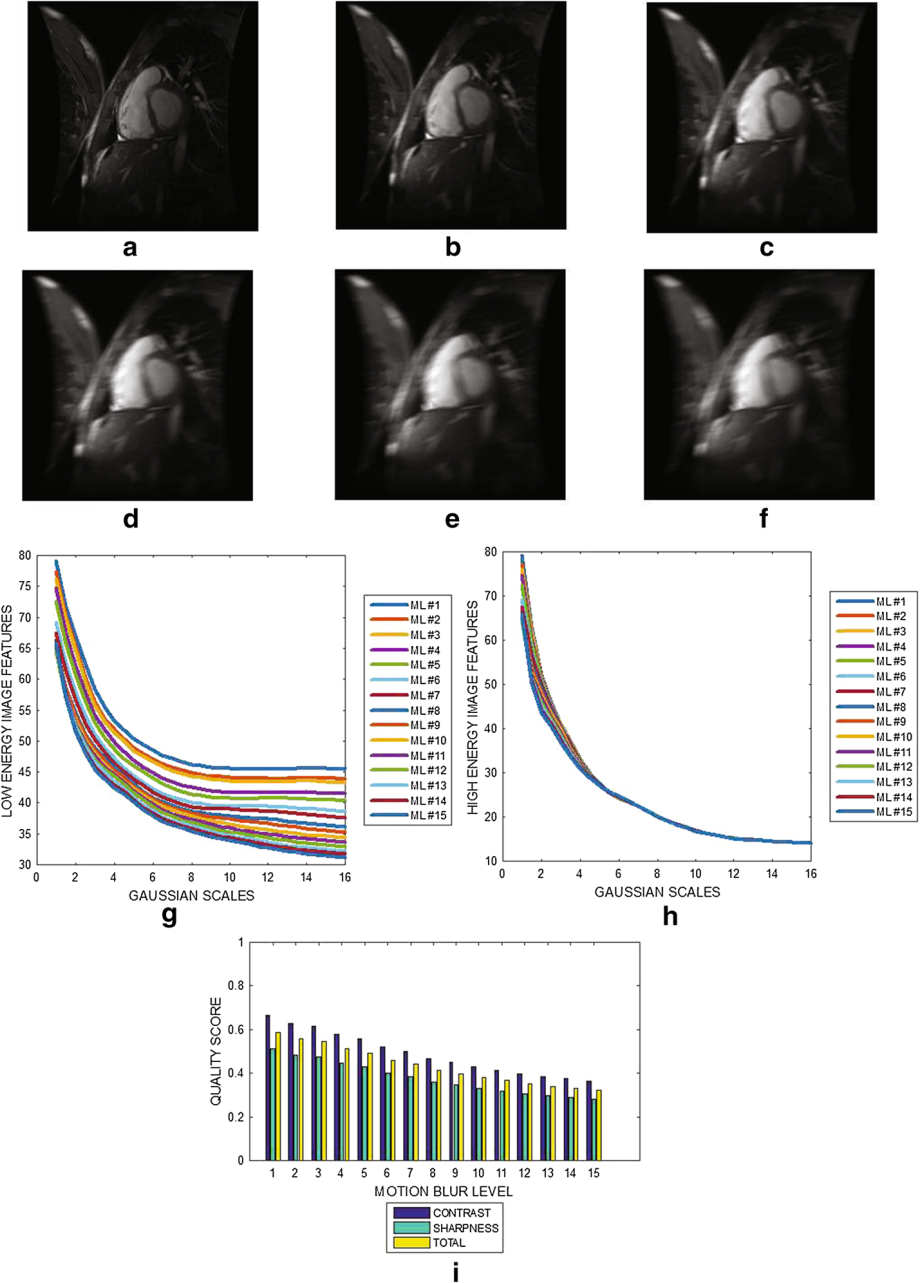
**a** Short axis cardiac MRI slice and its degraded versions at motion blur levels **b** 4, **c** 7, **d** 10, **e** 13 and **f** 15, **g** variation of image features of each slice from the output of the low energy Gaussian filter at different Gaussian scales. **h** Variation of image features of each slice from the output of the high energy Gaussian filter at different Gaussian scales. **i** Contrast, sharpness and total quality scores for different levels of motion blur

Figure 6a is a slice from cardiac MRI volume data. The images in Fig. 6b–f are the same image in Fig. 6a but were blurred with Gaussian filter at levels 4, 7, 10, 13 and 15, respectively. Given specific Gaussian blur, the variation of the image features at different Gaussian scales for the low and the high energy DOG filters are displayed in Fig. 6g, h, respectively. The contrast, sharpness and the total quality scores for Gaussian blur levels from 0 to 15 are shown in Fig. 6i. There is ≈ 50% decrease in the predicted quality index as the blur level increase from 0 to 15.

#### Motion blur

**Fig. 7:**
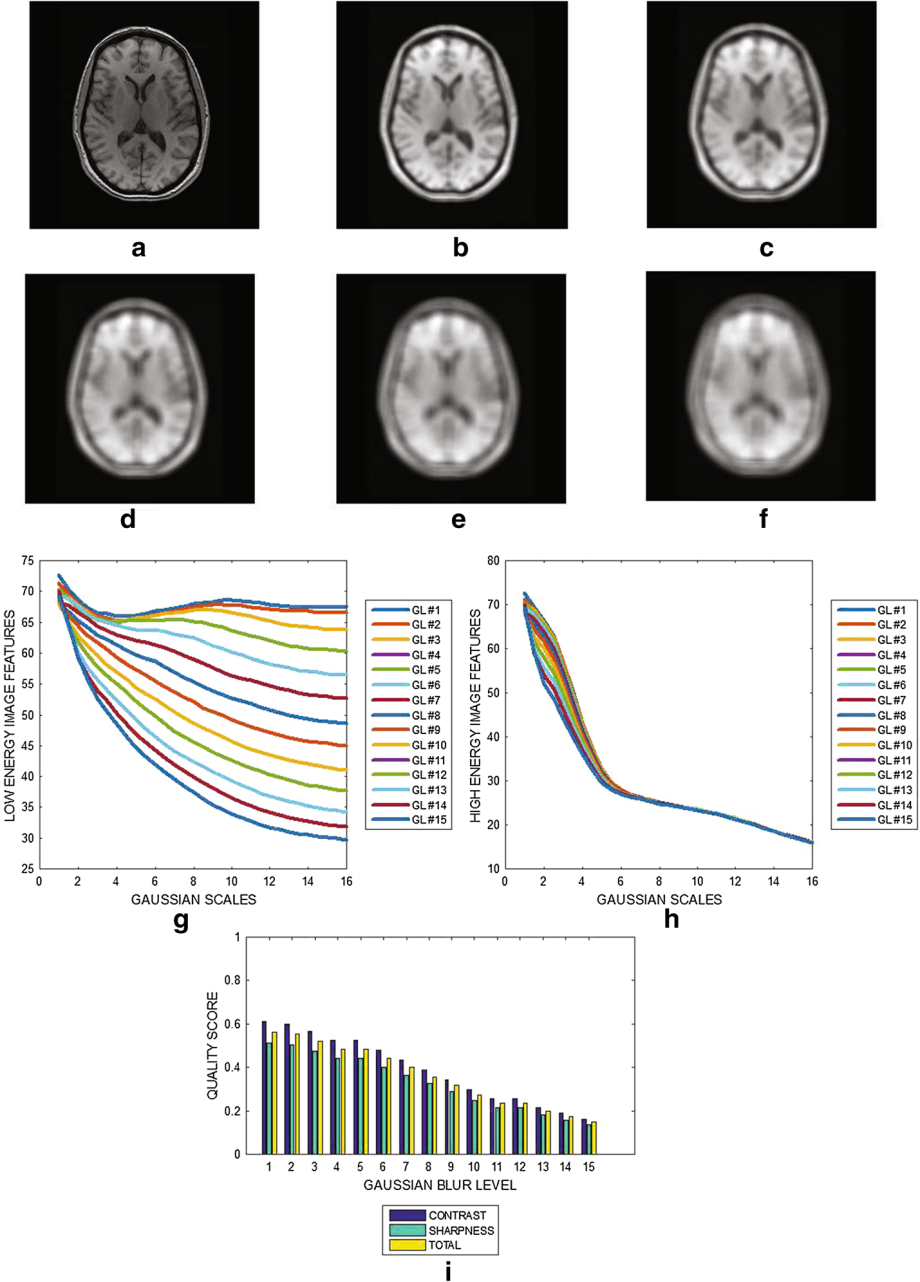
**a** Conventional T1 brain MRI slice from NeuroRx and its degraded versions at Gaussian blur levels **b** 4, **c** 7, **d** 10, **e** 13 and **f** 15, **g** variation of image features of each slice from the output of the low energy Gaussian filter at different Gaussian scales. **h** Variation of image features of each slice from the output of the high energy Gaussian filter at different Gaussian scales. **i** Contrast, sharpness and total quality scores for different levels of Gaussian blur

Figure 7a is a conventional T1 weighted brain MRI slice. Its motion blurred versions for motion blur levels of 4, 7, 10, 13 and 15 are shown in Fig. 7b–f, respectively. Variation of the image features for different Gaussian scales for the low and the high energy DOG filters are displayed in Fig. 7g, h, respectively. The contrast, sharpness and the total quality scores for motion blur levels from 0 to 15 are displayed in Fig. 7i. The predicted quality scores deceases from ≈ 0.6 to ≈ 0.15 for motion blur level which increased from 1 to 15.

**Fig. 8:**
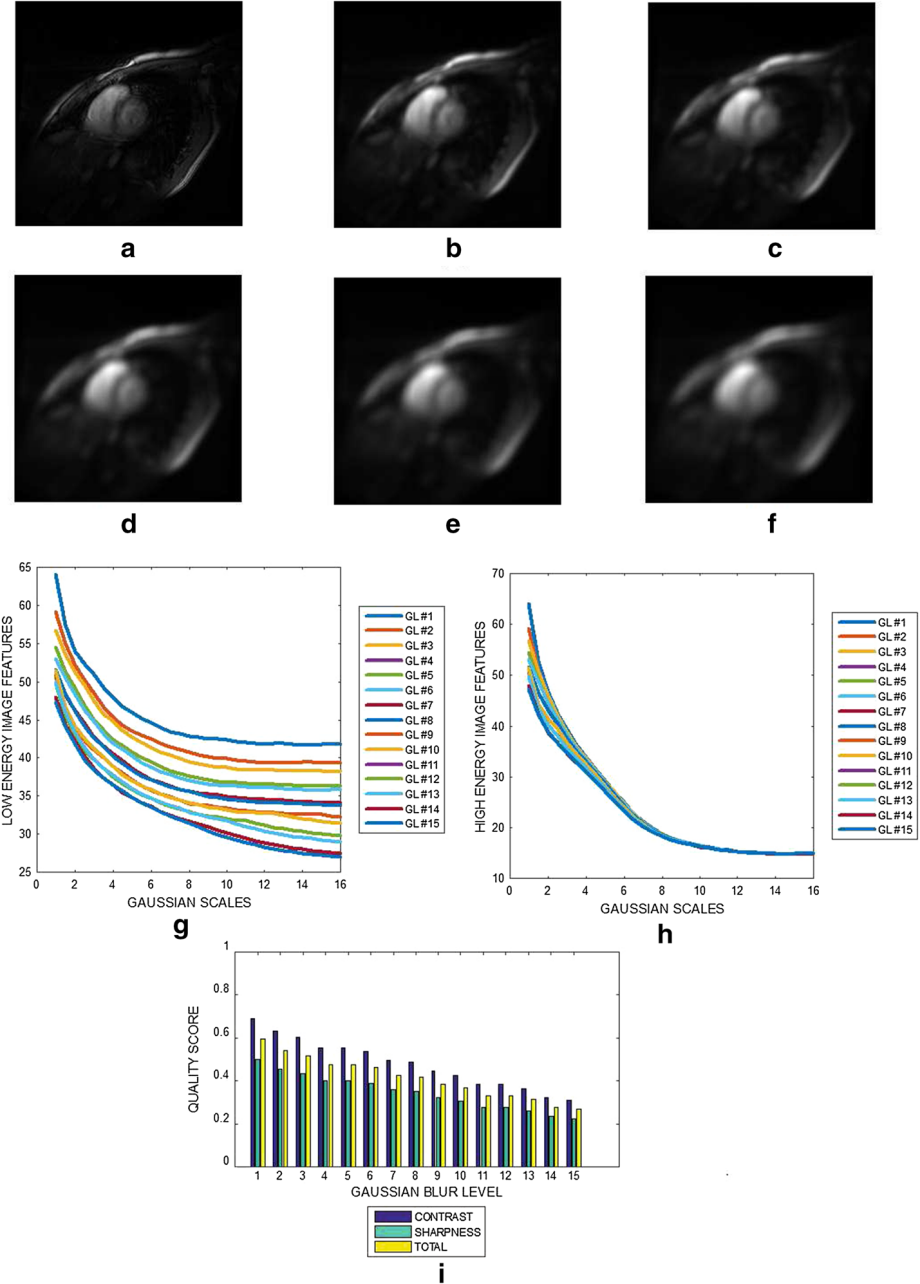
**a** Short axis cardiac MRI slice and its degraded versions at Gaussian blur levels **b** 4, **c** 7, **d** 10, **e** 13 and **f** 15, **g** variation of image features of each slice from the output of the low energy Gaussian filter at different Gaussian scales. **h** Variation of image features of each slice from the output of the high energy Gaussian filter at different Gaussian scales. **i** Contrast, sharpness and total quality scores for different levels of Gaussian blur

A slice in a cardiac MRI volume data is shown in Fig. 8a. Its motion blurred versions are shown in Fig. 8b–f for motion blur levels of 4, 7, 10, 13 and 15, respectively. The variation of the image features for different Gaussian scales for the low and the high energy DOG filters are displayed in Fig. 8g, h. The plot of the motion blur levels from 0 level to 15 level versus contrast, sharpness and the total quality scores are displayed in Fig. 8i. The quality scores decrease from 0.6 to 0.3 for motion blur level which increase from 1 to 15.

### Subjective validation

Results from the subjective evaluation of our proposed method are tabulated in Tables 1, 2, 3, 4, 5, 6, 7 and 8. Tables 1, 2, 3, and 4 are the results for cardiac, T2, conventional T1 and FLAIR brain MRI volume data degraded by motion blur. Corresponding results for degradation by Gaussian blur are displayed in Tables 5, 6, 7 and 8.

**Table 1:** Results from validation studies for short axis cardiac MRI volume data degraded by motion blur

Motion blur degradation level	Number of slices	Average objective score	Average subjective scores	Correlation coefficient
0	1200	0.70	0.73	0.80
5	1200	0.61	0.65	0.80
10	1200	0.40	0.45	0.75
15	1200	0.30	0.4	0.71

**Table 2:** Results from validation studies for T2 brain MRI volume data degraded by motion blur

Motion blur degradation level	Number of slices	Average objective score	Average subjective scores	Correlation coefficient
0	500	0.85	0.8	0.85
5	500	0.78	0.75	0.80
10	500	0.45	0.50	0.75
15	500	0.4	0.45	0.70

**Table 3:** Results from validation studies for conventional T1 brain MRI volume data degraded by motion blur

Motion blur degradation level	Number of slices	Average objective score	Average subjective scores	Correlation coefficient
0	250	0.75	0.73	0.78
5	250	0.70	0.65	0.75
10	250	0.40	0.42	0.72
15	250	0.35	0.38	0.68

**Table 4:** Results from validation studies for FLAIR brain MRI volume data degraded by motion blur

Motion blur degradation level	Number of slices	Average objective score	Average subjective scores	Correlation coefficient
0	300	0.68	0.70	0.75
5	300	0.63	0.65	0.75
10	300	0.43	0.40	0.70
15	300	0.35	0.30	0.70

**Table 5:** Results from validation studies for short axis cardiac MRI volume data degraded by Gaussian blur

Gaussian blur degradation level	Number of slices	Average objective score	Average subjective scores	Correlation coefficient
0	1200	0.70	0.73	0.80
5	1200	0.60	0.60	0.70
10	1200	0.45	0.42	0.70
15	1200	0.40	0.35	0.65

**Table 6:** Results from validation studies for T2 brain MRI volume data degraded by Gaussian blur

Gaussian blur degradation level	Number of slices	Average objective score	Average subjective scores	Correlation coefficient
0	500	0.85	0.80	0.85
5	500	0.75	0.75	0.81
10	500	0.40	0.45	0.75
15	500	0.40	0.35	0.70

**Table 7:** Results from validation studies for conventional T1 brain MRI volume data degraded by Gaussian blur

Gaussian blur degradation level	Number of slices	Average objective score	Average subjective scores	Correlation coefficient
0	250	0.75	0.73	0.78
5	250	0.72	0.70	0.73
10	250	0.42	0.45	0.70
15	250	0.35	0.35	0.70

**Table 8:** Results from validation studies for FLAIR brain MRI volume data degraded by Gaussian blur

Gaussian blur degradation level	Number of slices	Average objective score	Average subjective scores	Correlation coefficient
0	300	0.68	0.70	0.85
5	300	0.65	0.60	0.73
10	300	0.40	0.42	0.70
15	300	0.35	0.35	0.68

Tables 1, 2, 3 and 4 shows that for motion blur level which increase from 0 to 15, observers agreement decrease, from 0.80 to 0.71, from 0.85 to 0.70, from 0.78 to 0.68 and from 0.75 to 0.70, for cardiac, T2, conventional T1 and FLAIR brain MRI volume data, respectively. Corresponding results for Gaussian blur as shown in Tables 5, 6, 7 and 8 are from 0.80 to 0.65, from 0.85 to 0.70, from 0.78 to 0.70 and from 0.85 to 0.68.

## Discussion

Edge information is highly desired in medical images because it can potentially reveal details on the structures associated with normal anatomy and various pathological conditions [13]. The proposed blur assessment method predict the level of blur distortion in an image by generating and analyzing an edge map.

An important characteristics of the proposed method is its standardized quality index. The quality index lies between 0, the quality index for an extremely degraded image and 1, the quality index for an ideal image. The standardized quality index makes the algorithm suitable for application in large clinical trials for evaluating and comparing MRI images acquired from different scanners and different clinical trial sites.

The results displayed in Figs. 3 and 4 demonstrate that the proposed algorithm can assess the variations in the level of blur in the different slices contained within an MRI slice. The criteria for the diagnosis of MS lesions include the presence of periventricular and juxtacortical lesions which are located by the boundary between different brain tissues. Performance evaluation results show that our proposed method will be useful in the clinical trials to assess the reliability of edge information contained in the MRI data.

The plots displayed in Figs. 3, 4, 5, 6, 7 and 8 show general decrease in the contrast and sharpness quality scores for increasing levels of blur. This is a clear evidence that our proposed method can fairly compare and discriminate images based on their levels of blur.

The subjective evaluation results shown in Tables 1, 2, 3, 4, 5, 6, 7 and 8 is evidence that the multiscale representation effectively incorporates HVS characteristics in our proposed method. In all the categories of the experiment there is very good correlation between the objective scores predicted by our proposed method and the subjective evaluation assigned by human observers. The minimum and the maximum correlation coefficient is 0.65 and 0.85, respectively.

## Challenges, limitations and future work

Two major challenges may limit the accurate prediction of quality scores. The first is accurate segmentation of the foreground. Inaccurate segmentation can result in wrong computation of image features such as the mean of the test image and the mean of the edge map. If the foreground region is underestimated or overestimated the blur quality index will not correlate with the perceptual quality index. The second challenge is the sensitivity of the algorithm to the size of filter. Future work will focus on how to optimize the size of filter for different dimensions of the image. We hope to incorporate segmentation algorithm so that the algorithm can output blur assessment index for local regions within a slice. This approach will make the algorithm suitable for blur assessment in pathological conditions such as focal cortical dysplasia.

## Conclusion

This report propose a new approach to assess the blur level in a MRI image. The proposed method is based on the concept that the quality of an image is measured from the existence and persistence of structural information at different Gaussian scales. The contrast and sharpness features in the image are extracted by simultaneously convolving the image with two multiscale difference of Gaussian filters. The multiscale difference of Gaussian filters extract edge information from the test image and also incorporates human visual system characteristics into the algorithm. The parameters of each difference of Gaussian filter is tuned to either highlight or erode edges. After the conclusion of multiscale representation, blur level is assessed from the difference between the contrast and sharpness quality features in the images at the output of each filter.

The proposed method was evaluated on cardiac and brain MRI images and validated subjectively using human observers. Performance evaluation shows that the proposed method addresses most of the drawbacks associated with current blur assessment methods for MRI images. The quality prediction which lies between 0 and 1 makes it possible to compare the quality scores for different images and images with different contents. Features extracted from the test image are the first moments. This makes the algorithm computationally efficient. The blind nature of the proposed method coupled with computational efficiency makes the proposed method suitable for automated environments and different applications such as clinical trials where large volumes of data are processed.

## Abbreviations

MRI: magnetic resonance imaging
DOG: difference of Gaussian
LCM: local contrast feature
ADNI: Alzheimer’s disease neuroimaging initiative
FCD: focal cortical dysplasia
T2: transverse relaxation
T1: longitudinal relaxation
FLAIR: fluid attenuated inversion recovery
